# A Laser-Induced Mouse Model with Long-Term Intraocular Pressure Elevation

**DOI:** 10.1371/journal.pone.0107446

**Published:** 2014-09-12

**Authors:** Hongmin Yun, Kira L. Lathrop, Enzhi Yang, Ming Sun, Larry Kagemann, Valeria Fu, Donna B. Stolz, Joel S. Schuman, Yiqin Du

**Affiliations:** 1 Department of Ophthalmology, University of Pittsburgh School of Medicine, Pittsburgh, Pennsylvania, United States of America; 2 Louis J. Fox Center for Vision Restoration, University of Pittsburgh School of Medicine, Pittsburgh, Pennsylvania, United States of America; 3 Department of Cell Biology, Center for Biologic Imaging, University of Pittsburgh, Pittsburgh, Pennsylvania, United States of America; 4 McGowan Institute for Regenerative Medicine, University of Pittsburgh, Pittsburgh, Pennsylvania, United States of America; 5 Department of Bioengineering, Swanson School of Engineering, University of Pittsburgh, Pittsburgh, Pennsylvania, United States of America; 6 Department of Developmental Biology, University of Pittsburgh, Pittsburgh, Pennsylvania, United States of America; University of Melbourne, Australia

## Abstract

**Purpose:**

To develop and characterize a mouse model with intraocular pressure (IOP) elevation after laser photocoagulation on the trabecular meshwork (TM), which may serve as a model to investigate the potential of stem cell-based therapies for glaucoma.

**Methods:**

IOP was measured in 281 adult C57BL/6 mice to determine normal IOP range. IOP elevation was induced unilaterally in 50 adult mice, by targeting the TM through the limbus with a 532-nm diode laser. IOP was measured up to 24 weeks post-treatment. The optic nerve damage was detected by electroretinography and assessed by semiautomatic counting of optic nerve axons. Effects of laser treatment on the TM were evaluated by histology, immunofluorescence staining, optical coherence tomography (OCT) and transmission electron microscopy (TEM).

**Results:**

The average IOP of C57BL/6 mice was 14.5±2.6 mmHg (Mean ±SD). After laser treatment, IOP averaged above 20 mmHg throughout the follow-up period of 24 weeks. At 24 weeks, 57% of treated eyes had elevated IOP with the mean IOP of 22.5±2.5 mmHg (Mean ±SED). The difference of average axon count (59.0%) between laser treated and untreated eyes was statistically significant. Photopic negative response (PhNR) by electroretinography was significantly decreased. CD45+ inflammatory cells invaded the TM within 1 week. The expression of SPARC was increased in the TM from 1 to 12 weeks. Histology showed the anterior chamber angle open after laser treatment. OCT indicated that most of the eyes with laser treatment had no synechia in the anterior chamber angles. TEM demonstrated disorganized and compacted extracellular matrix in the TM.

**Conclusions:**

An experimental murine ocular hypertension model with an open angle and optic nerve axon loss was produced with laser photocoagulation, which could be used to investigate stem cell-based therapies for restoration of the outflow pathway integrity for ocular hypertension or glaucoma.

## Introduction

**Glaucoma** is a major cause of blindness throughout the world and the second leading cause of irreversible blindness in the USA [1], [2]. Primary open angle glaucoma (POAG), the most common form of glaucoma, is characterized by a progressive optic neuropathy with loss of retinal ganglion cells (RGC) and optic nerve axons, resulting in impairment of visual function. An elevated intraocular pressure (IOP) is the most important risk factor for most forms of glaucoma including POAG. Experimental animal glaucoma models are normally generated by increasing IOP which reproduces many pathophysiological changes observed in human glaucoma patients [3].

The IOP is regulated primarily by a fluid resistance to the aqueous humor outflow [4]. The juxtacanalicular connective tissue (JCT) and Schlemm's canal endothelial cells are generally believed to be the major sites of resistance to the aqueous outflow, and hence the primary determinants of IOP [5]–[7]. Trabecular meshwork (TM) cells are also believed to have a major role in regulation of the aqueous outflow. For example, TM cells modulate the permeability of Schlemm's canal endothelial cells in vitro via paracrine signaling [8]–[11], and keep the aqueous outflow channels patent via phagocytic activities [12], [13]. Further, a decrease in TM cellularity was observed both in aged and glaucomatous eyes [14]–[22]. The presence of outflow tract pathologies and role of IOP elevation in glaucoma justify the use of ocular hypertensive animal models for the study of RGC damage without ancillary injury to the retina and ocular structures [23]. Laser photocoagulation of the distal outflow pathway is frequently used to elevate IOP in animal models. Levkovitch-Verbin et al [24] reported the translimbal laser photocoagulation approach to create a rat glaucoma model. Since then, many groups have induced ocular hypertension in animal models by using laser photocoagulation of the anterior angle and episcleral veins [3], [25]–[36]. Aihara et at [25] showed that laser on mouse eyes with pupil dilation and anterior chamber flattening induced significant IOP increase with completely closed angle. Fu and Sretavan [29] applied laser on limbal and episcleral veins of albino mice and the IOP was doubled within 4 hrs but returned to normal by 7 days. They claimed [30] that, in C57BL/6 mice, no consistent and reproducible results with laser-induced ocular hypertension were obtained.

Using stem cells to repopulate the TM in glaucomatous eyes is one of the potential therapy strategies for POAG. We have already successfully isolated and characterized stem cells from human TM [37] and proved that these stem cells have the ability to home to the TM region after being transplanted into normal mouse anterior chamber [38]. In the present study, we developed a mouse model with IOP elevation by damaging the TM with laser photocoagulation. We characterized the effects of the TM damage to determine the model's suitability to investigate the potential of stem cell-based therapies for the outflow pathway reconstruction for glaucoma.

## Materials and Methods

### Materials

Antibodies used include anti-CD-45-PE conjugated (BD Pharmingen, San Diego, CA) and SPARC (R&D Systems, Minneapolis, MN). Goat anti-mouse IgG Alexa-488 secondary antibody, Alexa Fluor 633 phalloidin and 4′, 6-diamidino-2-phenylindole (DAPI) were purchased from Life Technologies (Carlsbad, CA). TUNEL assay kit (In Situ Cell Death Detection Kit, TMR red) was from Roche Molecular Biochemicals (Indianapolis, IN).

### Animals

Healthy adult female and male C57BL/6 mice aged from 4-week to 2-year old were purchased from Charles River Laboratories International, Inc. (Wilmington, MA). Mice were maintained in the University of Pittsburgh Animal Facility with low-light conditions and a 12-hour light-dark cycle and free access to food and water. All experimental procedures were reviewed and approved by the University of Pittsburgh Institutional Animal Care and Use Committee and handled according to guidelines provided in the Association for Research in Vision and Ophthalmology Resolution on the Use of Animals in Ophthalmic and Vision Research.

**IOP** was measured under the housing low-light conditions in the afternoons at approximately the same time using a rebound tonometer for rodents (TonoLab; Colonial Medical Supply, Franconia, NH) on mice that had been anesthetized by intraperitoneal injection of 2 mg of ketamine hydrochloride and 0.2 mg of xylazine (IVX Animal Health, Inc., St. Joseph, MO) mixed in 0.2 ml of Dulbecco's phosphate buffered saline (PBS). Baseline IOP was measured prior to the laser treatment.

**Laser photocoagulation** was performed on 8–12 weeks old C57BL/6 female mice using a 532 nm cw-diode laser (OcuLight GL Diode Laser, IRIDEX) at 80-mW laser power, 150-ms duration and 50-µm diameter spot size determined after preliminary studies with different combinations of the parameters for laser treatment. Approximately 25 laser burns were delivered at the gray zone of the limbus of each mouse. A second laser treatment was performed one week after. IOP was measured two to three times a week for the first 3 weeks after laser and once a week thereafter.

In this study, 50 eyes were treated with laser and mice were sacrificed at 1 day, 1 week, 2 weeks and 12 weeks after the second laser treatment for histology and transmission electron microscopy (TEM). Three pairs of optic nerves with and without laser treatment were collected for toluidine blue staining to quantify the optic nerve axon damage.

**Electroretinography (ERG)** was performed on both treated and untreated eyes at 20 weeks following laser treatment using an Espion Diagnosys system (Diagnosys LLC, Littleton, MA). After overnight dark adaptation (minimum 12 hours), mice were prepared for ERG recording under dim red illumination. Pupil dilation and topical anesthesia were achieved by topical application of 1 drop each of 0.5% tropicamide (Akorn, Lake Forest, IL) and 0.5% proparacaine hydrochloride (Falcon Pharmaceuticals, Fort Worth, TX). Body temperature was maintained at 37°C with a homeothermic controller and unit. Electrical signals were recorded with two 3-mm gold wire loop electrodes (Diagnosys) contacting the corneal surface of eyes precoated with a 2.5% hydroxypropyl-methylcellulose solution (Gonak, Akorn). A subdermal needle electrode (Viasys Healthcare, Chicago, IL) between the ears served as common reference while the other subdermal needle electrode inserted at the base of the left leg acted as ground. Retinal responses were recorded simultaneously from both eyes. Light stimuli were delivered via a ColorDome unit. Three different stimulus strengths of 1, 5 and 7 cd.s/m^2^ were used in a 3-step examination. In each step, the stimulus frequency was 2 Hz with 4 ms of on time green light on a blue background with the intensity of 10 cd/m^2^. At each intensity, 50 sweeps were recorded with sample frequency at 1 kHz, sweep pre-trigger time of 10 ms and sweep post-trigger time of 150 ms.

Data were analyzed using Espion software (Diagnosys LLC). Amplitudes of the a-wave, b-wave and photopic negative response (PhNR) were measured by identifying the maximum peak and trough and obtaining the baseline trough and peak amplitude, and then by taking the amplitude at a fixed criterion time after the stimulus onset, again with respect to baseline.

In vivo imaging of mouse anterior segments was performed using a high-resolution stereo fluorescence biomicroscope with vertical fluorescence illuminator (Leica MZFLIII; Leica Microsystems Inc., Bannockburn, IL). Mice were anesthetized and immobilized with a three-point stereotactic mouse restrainer as previously described [39], [40]. Images were obtained at a magnification of 25× with visibility to the limbus, anterior chamber, iris, pupil, and lens.

**Spectral-domain optical coherence tomography (SD-OCT)** scanning was adapted from the procedures described previously [41], [42]. Before each session, mice were anesthetized with an intraperitoneal injection of ketamine and xylazine to prevent large movements during SD-OCT image acquisition. Mice were secured on a custom stage that allowed for free rotation to acquire images focusing on the cornea. Images centered on the cornea were acquired using SD-OCT (Bioptigen, Inc., Research Triangle Park, NC). All SD-OCT images consisted of a 250×250 A-scan array; there were 250 A-scans per B-scan, 250 B-scan frames, and 1024 samplings/A-scan in depth.

### Histology

For plastic sectioning, enucleated mouse eyeballs were fixed in 2% glutaraldehyde in 0.1 M PBS at room temperature for 2 hours followed by post-fixation in 10% formalin at room temperature overnight. Samples were embedded in plastic (JB-4, Electron Microscopy Sciences, Hatfield, PA), sectioned for 2- µm thickness and stained with hematoxylin and eosin.

All other procedures were carried out as described previously [40]. In brief, enucleated mouse eyeballs were fixed in 1% paraformaldehyde at 4°C overnight followed by either storage at 4°C in 50% glycerol and 50% PBS (v/v) for wholemount staining or frozen at −20°C in optimal cutting temperature embedding compound (Tissue-Tek OCT; Electron Microscopy Sciences, Hatfield, PA) and cut into 9-µm thick cryosections on a cryostat for immunofluorescence. Sections were hydrated in PBS and postfixed in 2% paraformaldehyde for 15 minutes. Nonspecific binding was blocked with 10% heat-inactivated goat serum and anti-mouse CD16/CD32 Fcγ III/II (BD Pharmingen). Sections were incubated with primary antibodies overnight at 4°C. After two rinses in PBS, secondary antibodies and DAPI were added for 1 hour at room temperature. Images were acquired on a confocal microscope with a 40× objective (Olympus FluoView FV1000 confocal microscope; Olympus, Center Valley, PA).

#### Wholemount Stain

After fixation, eyes were cut 1.5 mm posterior to the limbus. The anterior part, including the cornea and TM, was cut into quarters for wholemount stain. The iris was carefully removed before staining. Nonspecific binding was blocked as described above. The tissue was incubated with anti-CD45 antibody conjugated PE and phalloidin-633 (stains F-actin) overnight at 4°C. Following five washes, the tissue was incubated with DAPI for 10 min at room temperature prior to confocal imaging. Stitched image stacks were acquired by sequential scanning to avoid fluorescence crossover on a confocal microscope (Olympus).

**TUNEL** assay was performed using a cell death detection kit (In Situ Cell Death Detection Kit) following the manufacturer's protocol on cryopreserved tissue. Nuclei were stained with DAPI. At least three independent TM tissues from each condition and eight sections of each condition were stained and imaged using a confocal microscope.

### Transmission Electron Microscopy (TEM)

The ultrastructure of mouse TM was examined by TEM. Mouse eyeballs were fixed in cold 2.5% glutaraldehyde (EM grade, Taab Chemical) in 0.1 M PBS pH 7.3, rinsed in PBS, and were then post-fixed in 1% osmium tetroxide (Electron Microscopy Sciences) with 1% potassium ferricyanide (Fisher). They were dehydrated through a graded series of ethanol baths and embedded in Epon (made from dodecenyl succinic anhydride, nadic methyl anhydride, Scipoxy 812 Resin and 2,4,6-tris(dimethylaminomethyl)phenol, Energy Beam Sciences). Semi-thin (300 nm) sections were cut on a Reichart Ultracut, stained with 0.5% toluidine blue (Fisher) and examined under a light microscope. Ultrathin sections (65 nm) stained with uranyl acetate (Electron Microscopy Sciences) and Reynold's lead citrate (Fisher) were examined and photographed at 80 kV on a Jeol 1011 transmission electron microscope.

### Mouse Optic Nerve Axon Assessment

Optic nerves were dissected from 1 mm behind the eyes to the optic chiasm. Fixation followed the same procedures that were used for TEM. The proximal part of the optic nerve was cross-sectioned at 0.5 µm and stained with 1% toluidine blue. Images were acquired using light microscopy with a BX60 microscope (Olympus) equipped with a Spot digital camera (Diagnostics Instruments, Inc., Sterling Heights, CA). For each nerve, at least five 60× fields were acquired. Axon number counts and average axon number per µm^2^ were analyzed by a masked observer using ImageJ software (National Institute of Health, Bethesda, MD).

### Statistical Analysis

All values are presented as mean ±SD or mean ±SEM. The statistical differences were determined by paired *t*-test to assess the significance of differences between two groups. Statistical significance was set at *p*<0.05. For IOP distribution on normal eyes, D'Agostino-Pearson normality test was performed.

## Results

### IOP of Normal C57BL/6 Mice Are Normally Distributed

**Figure 1:**
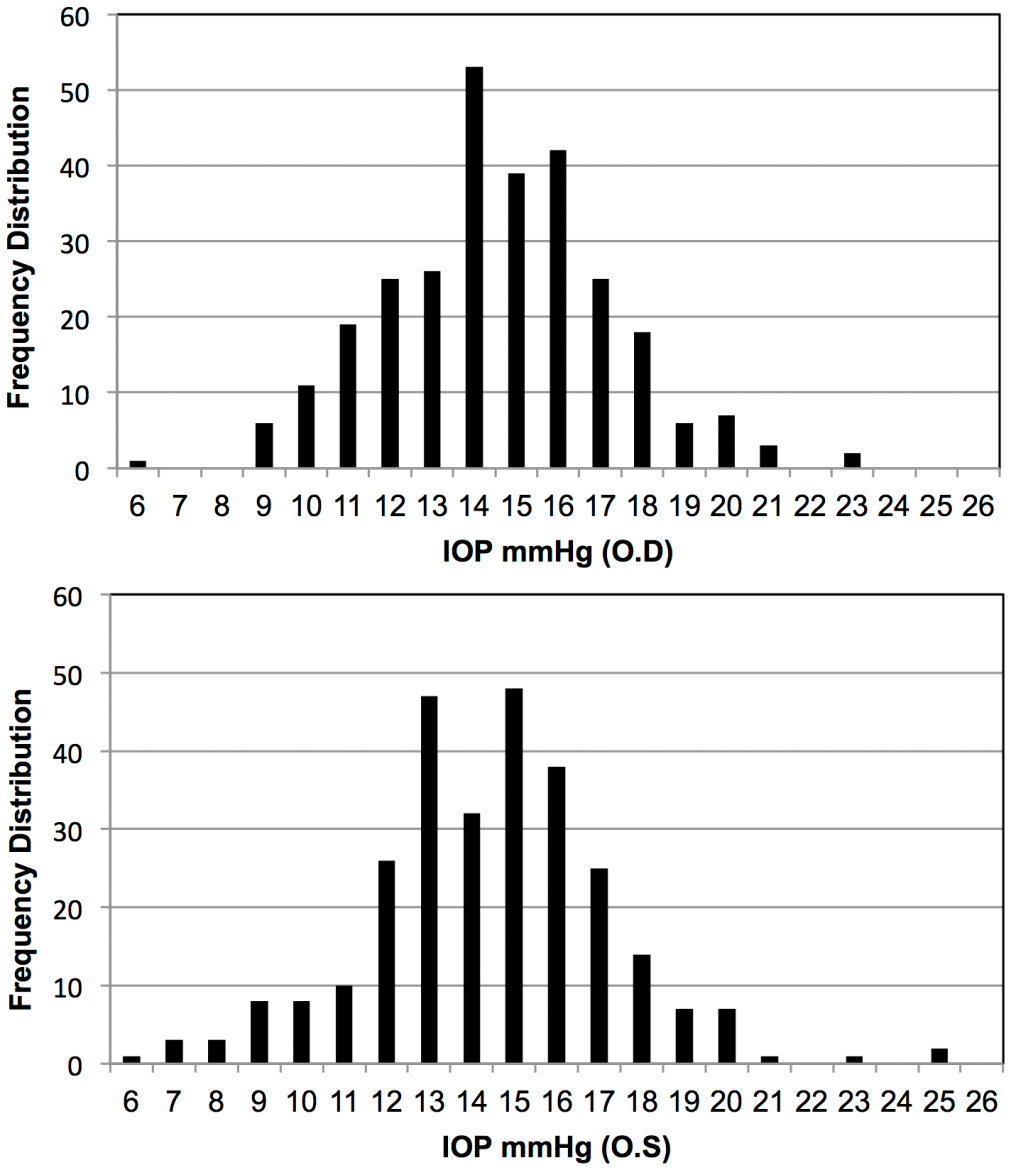
IOP range of normal C57BL/6 mouse population. IOP was measured on 281 healthy adult C57BL/6 mice under anesthesia. IOP of right (OD) and left eyes (OS) were recorded and analyzed separately. X-axis represents the IOP and y-axis represents the frequency distribution at different IOP levels.

The IOP of 281 healthy adult C57BL/6 mice of both sexes aged from 4-week to 2-year old was measured. The average IOP was 14.6±2.5 mmHg (mean ±SD, n = 281) in right eyes (OD) and 14.4±2.7 mmHg (mean ±SD, n = 281) in left eyes (OS) (Table 1). There was no statistically significant difference between two eyes (*p* = 0.2648, paired *t*-test). The IOP of both eyes are normally distributed (Fig 1) (*p* = 0.4735, D'Agostino-Pearson normality test) with 20 mmHg as the top 97.5th percentile and 20.3 mmHg as the top 99.5th percentile of normal IOP range in C57BL/6 mice.

**Table 1:** IOP distribution of normal C57BL/6 mice.

IOP (mmHg)	Mean	SD	95th percentile	97.5th percentile	99.5th percentile
OD	14.6	2.6	19	20	20.4
OS	14.4	2.7	19	20	20.2
OU	14.5	2.6	19	20	20.3

### Laser Photocoagulation to the TM Induces IOP Elevation

**Figure 2:**
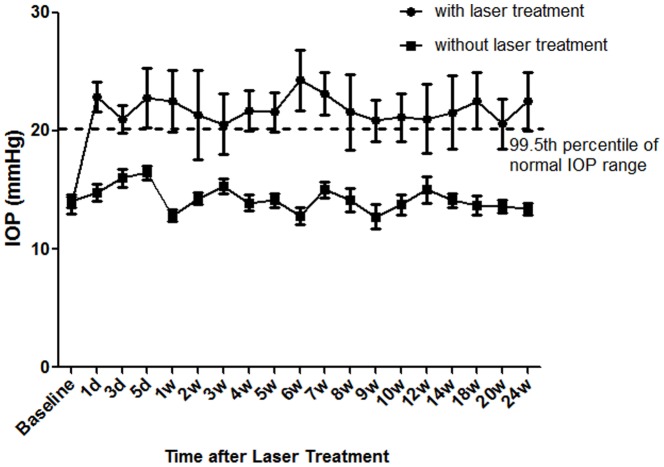
IOP elevation after laser treatment. IOP of laser treated and fellow untreated eyes was shown at different time points after laser. Values are mean ±SEM. Dashed line represents the 99.5th percentile of normal IOP range as shown in Table 1. The difference between two groups at each time points is statistically significant (*p*<0.05, paired *t*-test).

The IOP changes between laser-treated and untreated control eyes at different post-treatment time points are shown in Table 2 and Fig 2. The average IOP of laser treated eyes were above 20.3 mmHg, which is the top 99.5th percentile of normal IOP of C57BL/6 mice. In contrast, the average IOP of untreated eyes were below 20.3 mmHg. The IOP difference between treated and untreated eyes at each time point post-laser treatment was statistically significant (*p*<0.05, paired *t*-test).

**Table 2:** IOP changes at different time points after laser treatment.

Time Points	Eyes (n)	IOP(Mean ±SEM) (mmHg)	
		Laser treated	Untreated Controls
Baseline	50	13.7±0.7	14.1±0.5
1 day	50	22.9±1.2	14.8±0.7
1 wk	43	22.5±2.6	12.8±0.5
2 wks	36	21.3±3.8	14.3±0.5
12 wks	29	21.0±2.9	15.0±1.1
24 wks	22	22.5±2.5	13.4±0.5

**Figure 3:**
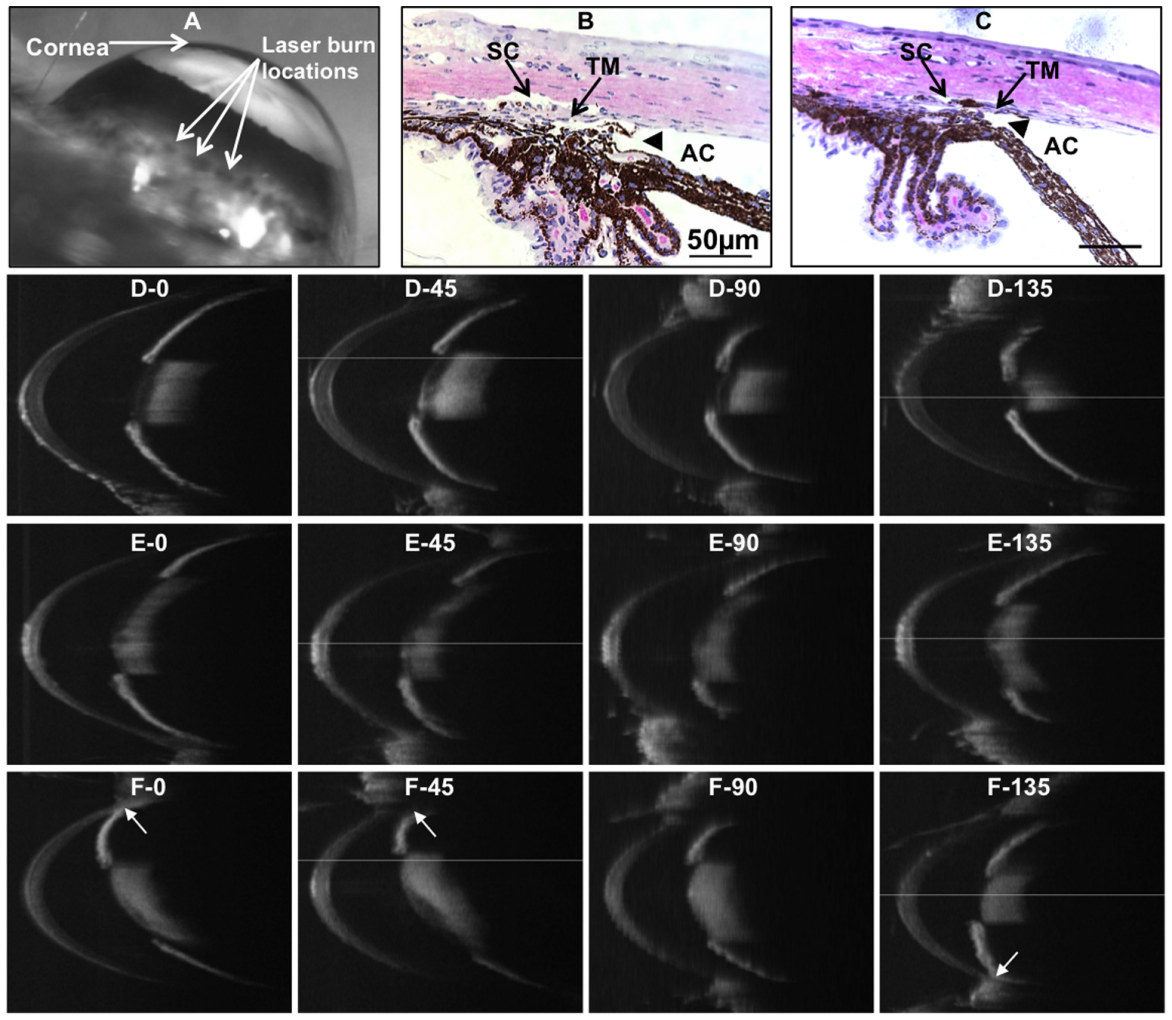
Mouse anterior chamber angle after laser treatment. A: Mouse eye at 4 weeks after laser treatment showing laser burn locations. B: Plastic section of a normal control eye with anterior chamber angle open (arrowhead). C: Plastic section of a laser-treated eye at 4 weeks with anterior chamber angle open (arrowhead). D–F: Representative images of SD-OCT scans. D: Normal control (IOP = 14 mmHg). E: Laser treated eye at 20 weeks (IOP = 30 mmHg) with anterior chamber angle open. F: Laser treated eye at 20 weeks with partial synechia (pointed by arrows, IOP = 28 mmHg). SD-OCT scans of each eye show the angles with rotations of the eye at 0, 45, 90 and 135 degrees. AC, anterior chamber. TM, trabecular meshwork. SC, Schlemm's canal. IOP, intraocular pressure.

The laser burns at the limbus were still visible at 4 weeks after laser treatment (Fig 3A), which indicates where the laser beams were shot through. Plastic sections show the anterior chamber angle of laser-treated eye remained open at 4 weeks after laser (Fig 3C), similar to that of normal control (Fig 3B).

SD-OCT scanning was used to detect the anterior chamber angle. The open-angle of normal eyes throughout 360 arc was shown as Figs 3D-0, D-45, D-90, D-135. Sixteen out of 18 laser-treated eyes that received OCT examination had open anterior chamber angles (Fig 3E-0, E-45, E-90, E-135) and only 2 eyes (11%) had partial synechia (<180°, Figs 3F-0, F-45, F-90, F-135).

Since the laser beams were controlled to be outside of the anterior chamber angle, hyphema was only observed in one eye out of 50 (2%) laser-treated eyes and no other side effects or complications were observed as a result of this treatment.

### IOP Elevation by Laser Photocoagulation Results in Optic Nerve Damage

**Figure 4:**
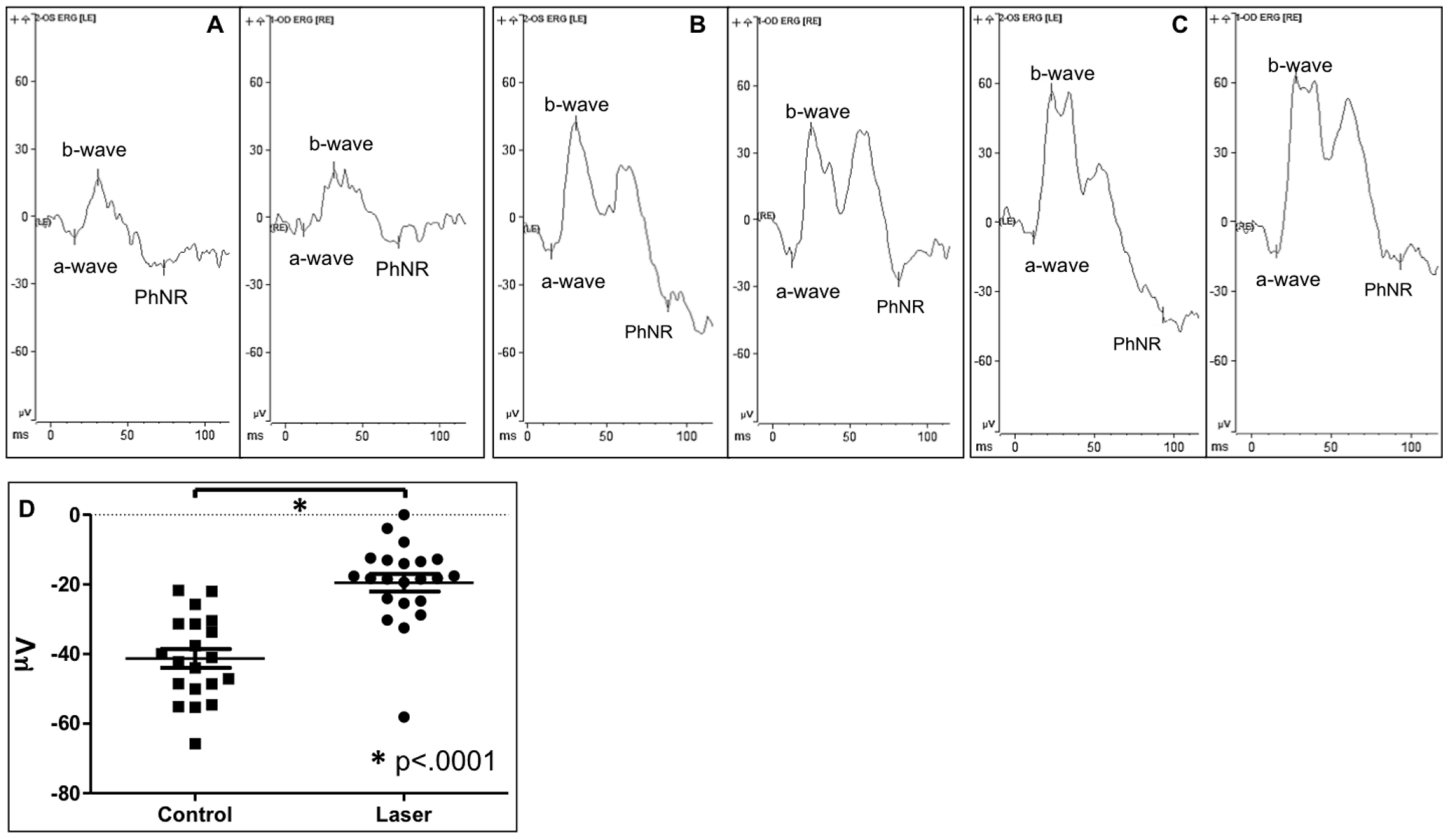
Photopic negative response (PhNR) of normal and laser-treated mouse eyes. A: a wave, b wave and PhNR of untreated left control eye (OS) and laser-treated right eye (OD) of step 1 with the stimulus at 1 cd.s/m^2^. B: Depicts the waves of step 2 with the stimulus at 5 cd.s/m^2^. C: Represents the waves of step 3 with the stimulus at 7 cd.s/m^2^. D: Shows the statistical analysis of the amplitude of PhNR at step 2 with the stimulus at 5 cd.s/m^2^ compared to the laser-treated and untreated control eyes (*p*<0.0001, paired *t*-test).

RGC function was evaluated using ERG. Fifty sweeps were recorded at 3 different stimulus strengths at 1 (step 1), 5 (step 2), and 7 cd.s/m^2^ (step 3) and the patterns of the PhNR changes at all three stimuli were about the same. Fig 4A, 4B and 4C are representative ERG waves under 3 different stimuli and the amplitude of PhNR of laser treated eye decreased comparing to the untreated contralateral eye, whereas the amplitudes of a-wave and b-wave did not show much difference between laser-treated and untreated eyes. Under stimulus of 5 cd.s/m^2^, the average amplitude of PhNR of the untreated control eyes was −41.29±2.70 µV (mean ±SEM) and that of laser treated eyes at 20 weeks was −19.50±2.51 µV (mean ±SEM). The difference was statistically significant (*p*<0.0001, paired *t*-test, Fig 4D).

**Figure 5:**
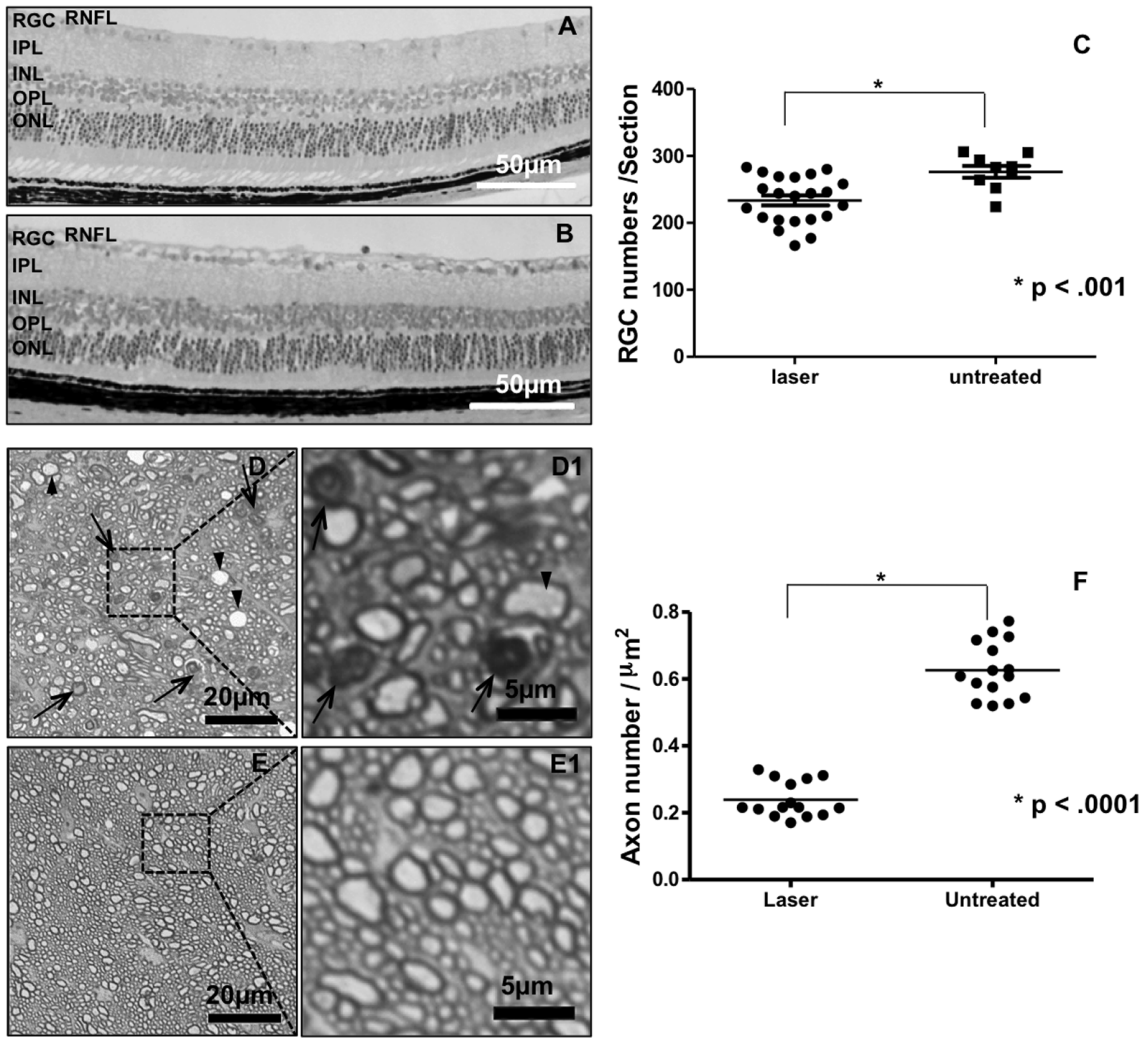
RGC and optic nerve axonal damage following IOP elevation. A and B represent images of plastic-embedded retinal sagittal sections peripheral to the optic nerve with hemotoxylin-eosin staining. A: Laser-treated. B: Normal control. C: Statistical analysis of the RGC number counts (*p*<0.001, paired *t*-test). D and E show toluidine blue staining of semithin transverse sections of optic nerves. D: Laser treated eye with swollen axons (arrowheads) and shrunken axons (arrows). E: Normal control with more homogeneous axons. D1–E1 are magnified views of the regions encased in black boxes in D–E. F Represents the statistical analysis of the axons (*p*<0.0001, paired *t*-test). RNFL, retinal nerve fiber layer; RGC, retinal ganglion cells; IPL, inner plexiform layer; INL, inner nuclear layer; OPL, outer plexiform layer; ONL, outer nuclear layer.

The characteristic of glaucomatous damage is the RGC loss while other retinal layers are relatively spared. Retinal histology at 24 weeks after laser treatment demonstrated prominent RGC cell loss and diminution of the retinal nerve fiber layer (RNFL) (Fig 5A) compared to normal controls (Fig 5B). The reduction of RGC number in laser-treated eyes compared to normal controls was statistically significant (p<0.001, paired *t*-test, Fig 5C). Toluidine blue staining on 0.5 µm optic nerve sections showed swollen (arrowheads) and shrunken (arrows) axons in the optic nerve of laser treated with IOP-elevated eyes (Fig 5D, D1) relative to the fellow untreated normal eyes (Fig 5E, E1). The axon numbers were counted semiautomatically using ImageJ and the axon numbers per µm^2^ were calculated. At least five 60× fields per nerve were counted. The average axon number per µm^2^ was reduced by 59.0% at 24 weeks in laser treated eyes compared to untreated eyes (Fig 5F) (0.07±0.01 axons/µm^2^ of eyes with laser treatment vs 0.18±0.02 (Mean ±SD) of untreated eyes, *p*<0.0001).

### Laser Induces Inflammatory Response and Fibrosis on the Outflow Pathway

**Figure 6:**
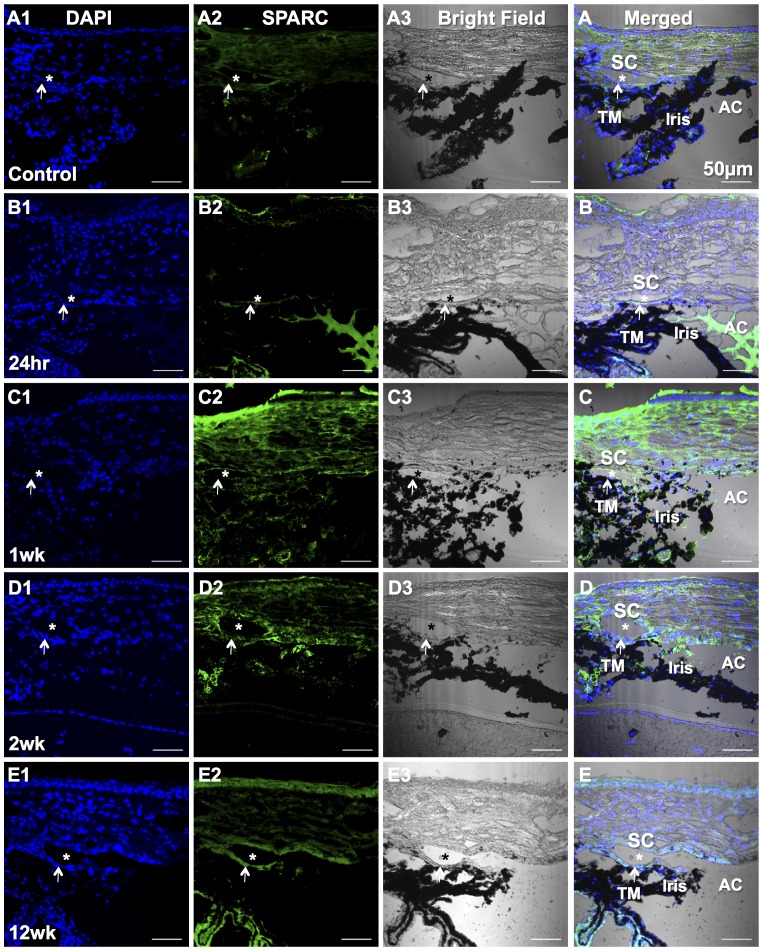
SPARC immunolocalization in the TM. A1–E1: DAPI stains nuclei as blue in the cryosections. A2–E2: SPARC stains the TM and peripheral cornea and sclera green. A3–E3: Bright field images for tissue orientation. A–E: Merged fluorescent images and bright field provide detailed structural orientation. Asterisk (*) shows the location of Schlemm's canal (SC). Arrow points the TM. Anterior chamber (AC) and iris are indicated as well. A: Untreated control. B: 24 hrs after laser treatment. C: 1 week after laser. D: 2 weeks after laser. E: 12 weeks after laser. Bars, 50 µm.

**Figure 7:**
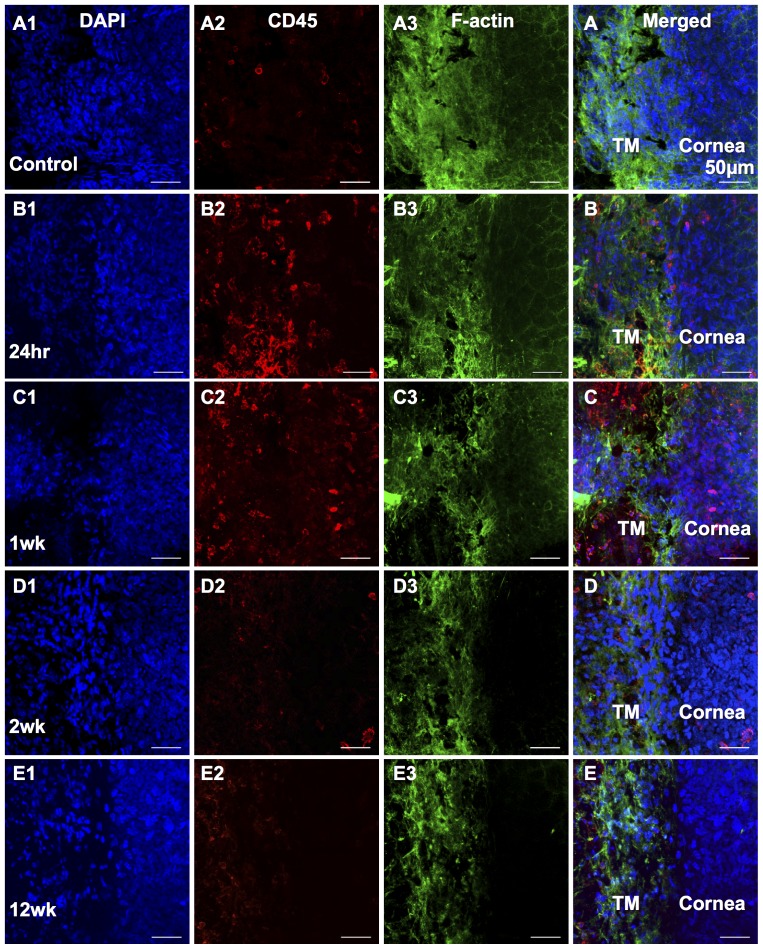
CD45 and F-actin immunolocalization in the TM wholemounts. A1–E1: DAPI stains nuclei as blue in the wholemounts. A2–E2: Inflammatory cells positive to CD45 (red) appear at 24 hrs after laser (B2) and decrease thereafter. A3–E3: F-actin (Green) stains the TM cells clearly shown the location of the TM region. A–E: Merged fluorescent images provide detailed structural orientation. The TM and cornea are indicated. A: Untreated control. B: 24 hrs after laser treatment. C: 1 week after laser. D: 2 weeks after laser. E: 12 weeks after laser. Bars, 50 µm.

Fig 6 shows SPARC staining on cryosections of normal control and eyes of 24 hrs, 1 week, 2 weeks, 12 weeks after laser treatment. SPARC expression was increased in the TM region as well as in the peripheral cornea and sclera at 1 week after laser treatment (Fig 6C) and it was remained in the TM region up to 12 weeks (Fig 6E). Laser treatment causes an obvious inflammatory response with anterior chamber infiltration in some eyes. Fig 6B shows SPARC staining in the anterior chamber indicating inflammatory infiltration at 24 hrs after laser. Wholemount staining (Fig 7) demonstrated that the expression of inflammatory cell marker CD45 peaked at 24 hrs (Fig 7B) after laser treatment in the TM region and was decreased at 1 week (Fig 7C) and continued to decline thereafter. There was almost no CD45 expression in the TM region at 12 weeks after laser (Fig 7E), similar to the normal control (Fig 7A). F-actin staining showed the localization of the TM (Fig 7A3–E3).

### TM Microstructure Changes are Related to IOP Elevation

**Figure 8:**
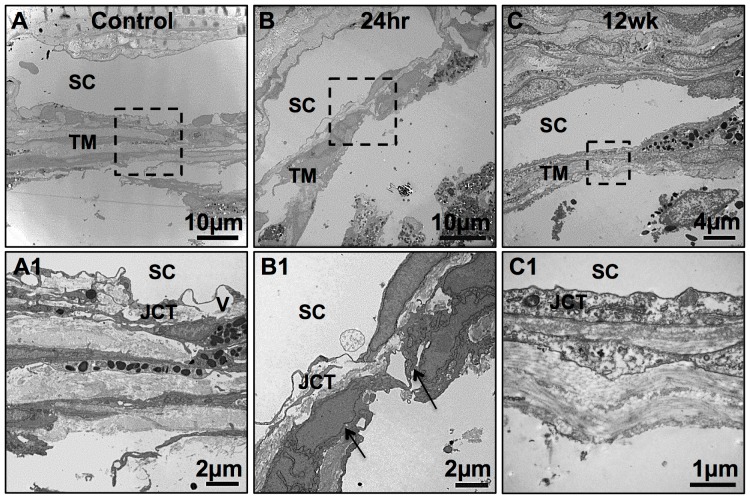
TM ultrastructure changes after laser. The region encased in the black box in the upper row is magnified in the lower row. A and A1: TEM of normal mouse TM revealed aligned collagen beams with three layers of normal TM cells. A giant vacuole (V) was seen. The JCT appeared loose and disorganized. B and B1: 24 hrs after laser, the TM tissue had only 1–2 layers of cells with disorganized beams. C and C1: 12 weeks after laser, the TM had disorganized beams and the collagen fibrils were more compacted compared to the normal structure and the JCT was more compacted with more and disorganized extracellular matrix. Abbreviations: SC, Schlemm's canal; JCT, Juctacanalicular connective tissue; V, giant vacuole.

The ultrastructure of mouse TM was examined by TEM. Fig 8A shows normal mouse TM containing trabecular beams with aligned collagen fibrils and three layers of normal TM cells. Giant vacuoles among the Schlemm's canal endothelial cells could be seen. The JCT appeared loose and disorganized compared to the organized trabecular beams in the TM. Twenty-four hrs after laser, there were only 1–2 layers of TM cells with some apoptotic without organelles (Fig 8B1, arrows). The beams were disorganized. Twelve weeks after laser, the TM tissue had more compacted JCT and collagen fibrils and disorganized ECM (Fig 8C, C1) compared to the normal structure (Fig 8A, A1). No giant vacuoles could be seen in the Schlemm's canal endothelial cells 12 weeks after laser (Fig 8C).

### Laser Photocoagulation Caused TM Cell Apoptosis

**Figure 9:**
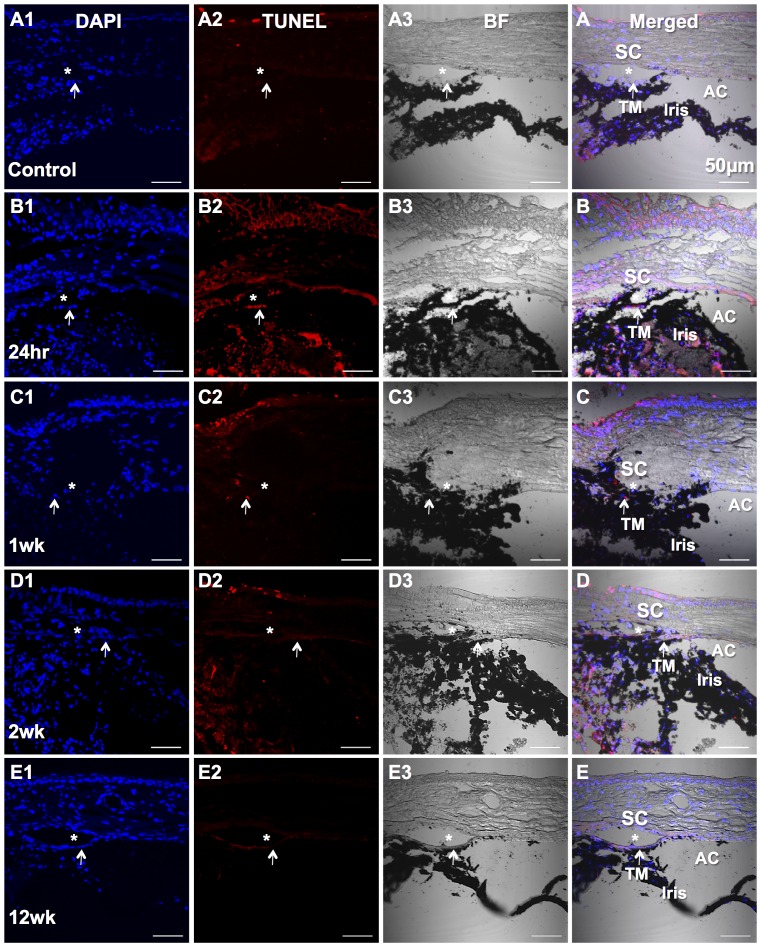
TUNEL staining on cryosections. A1–E1: DAPI stains nuclei as blue. A2–E2: TUNEL assay stains apoptotic cells as red peak at 24 hrs after laser (B2). A3–E3: Bright field for tissue orientation. A–E: Merged fluorescent images and bright field provide detailed structural orientation. Asterisk (*) represents the location of Schlemm's canal (SC). Arrow points the TM. Anterior chamber (AC) and iris are indicated as well. A: Untreated control. B: 24 hrs after laser treatment. C: 1 week after laser. D: 2 weeks after laser. E: 12 weeks after laser. Bars, 50 µm.

Apoptotic cells were detected in the corneal epithelium but not in the TM region in the normal control (Fig 9A) by TUNEL staining. There were many apoptotic cells in the TM region as well as the other tissues in the limbal region 24 hrs after laser treatment (Fig 9B). At 1 week, 2 weeks and 12 weeks after laser, less apoptotic cells were detected in the TM region (Figs 9C, D, E).

## Discussion

In the present study, we developed and characterized a mouse model using laser photocoagulation on the TM tissue through the limbus. IOP elevation lasted up to 24 weeks while maintaining an open anterior chamber angle and induced RGC damage demonstrated by PhNR changes and optic nerve axon loss. Laser photocoagulation elicited an acute inflammatory response and apoptosis in the TM region and induced fibrotic response and extracellular matrix (ECM) disorganization in the outflow pathway at later times. The IOP elevation presumably was induced by the structural changes other than mechanical blockage by anterior chamber angle synechia. This model may be suitable for stem cell-based therapies for remodeling and reconstructing the outflow pathway to maintain regular IOP and prevent optic nerve damage.

We measured the IOP of 281 healthy adult C57BL/6 mice and provided reliable documentation of normal IOP range and IOP distribution in C57BL/6 mice. 97.5th and 99.5th percentiles of the IOP of normal mice were determined. Clinically 99.5th percentile of the normal population has been used as the diagnostic threshold of pathogenic IOP when it is impossible to check the visual fundus and visual function [43]. The average IOP of laser treated eyes in this study at each time point after laser was above the 99.5th percentile. It has been reported that ketamine administration could affect mouse IOP and accurate IOP measurement in mice must be made within minutes [44]. By measuring IOP of normal mice immediately after anesthesia, we acquired a normally distributed IOP of normal mice and we concluded that the method we used to measure mouse IOP is reliable.

Many studies [3], [25]–[30] have worked on mice and rats to make glaucoma models using laser photocoagulation. They all focused on increasing IOP and inducing optic nerve damage but did not attempt to keep the normal morphology of the anterior segment and did not clarify the pathological changes of the outflow pathway and TM tissue. In this study, we treated the eyes specifically in several aspects. First, instead of dilating the pupils to cause mydriasis prior to laser treatment, we used 1% pilocarpine for miosis to open the angle. Grozdanic et al [28] also used pilocarpine in their study to increase outflow of photosensitive dye to the TM. Second, we did not drain the aqueous humor to flatten the anterior chamber. Third, we did not burn and block the episcleral veins. With less energy and smaller laser spot size, we successfully avoided the angle synechia which disturbs the TM structure and renders the model unsuitable for cell-based therapies for TM remodeling. Grozdanic et al [28] and Gunn et al [45] used similar parameters with 810 nm diode laser to build their high IOP animal models, but they either did not check the anterior chamber angle or found closed anterior chamber angle. Our data show that laser damage to the outflow pathway, even without mechanical blockage at the angle, can induce structural and hence functional changes to the outflow pathway to effectively elevate IOP for up to 24 weeks.

To confirm that the IOP elevation was caused by the outflow structural changes but not mechanical synechia, we used SD-OCT as well as histology to detect the anterior chamber angle. We show that among eighteen eyes with laser treatment, only two eyes had synechia of less than 180 arc and all the others had completely open anterior chamber angles (Fig 3). Hematoxylin-eosin staining on the plastic sections confirmed the open angles after laser treatment. Our mouse model thus presents one of the main characteristics, open anterior chamber angle, of human POAG, and may serve as an option for animal research on open angle glaucoma. Our data also indicate that SD-OCT is effective to examine the anterior chamber and the 360 degrees of the angle of mouse eye. Since OCT scanning can be done on live animals under anesthesia, it can be used to track the angle changes throughout the entire period of an experiment.

By definition, glaucoma is a group of diseases that damage the RGC and optic nerve. Pattern electroretinogram (PERG) is a well-established method to assess RGC function [46], [47]. PhNR, a component of the full-field ERG, is another method to assess RGC function in glaucoma patients since 1999 [48], [49]. Both PhNR and PERG are nearly equal in detecting early glaucoma in human patients [50]. The PhNR component of the full-field ERG can be recorded in mice and is sensitive to elevation of IOP [51]. In this mouse model, we demonstrate that PhNR amplitude of eyes with IOP elevation caused by laser photocoagulation was reduced dramatically (Fig 4), consistent with the RGC loss and optic nerve damage (Fig 5). On the other hand, the outer layers of retina were not damaged by laser treatment (Fig 5A), which is consistent with the ERG results that a-wave and b-wave of the ERG remained similar between the laser-treated eyes and contralateral untreated eyes (Fig 4).

Aging produces a decreased TM cellularity and additional cellular loss beyond that of normal aging has been found in the TM of human glaucomatous eyes [14]–[22]. The ECM of the TM is thought to be important in regulating IOP in both normal and glaucomatous eyes. The ECMs of the TM beams, JCT and Schlemm's canal inner wall are comprised of fibrillar and non-fibrillar collagens, elastin containing microfibrils, matricellular and structural organizing proteins, glycosaminoglycans and proteoglycans. The ECM of outflow pathway is relatively dynamic, undergoing constant turnover and remodeling [4]. F-actin architecture in the TM cells responds to the local environment changes [52]–[54]. With laser photocoagulation, we show that the TM cell layers were decreased (Fig 8) and the ECM of the TM and JCT became compacted and disorganized (Fig 8).

SPARC (secreted protein, acidic and rich in cysteine) is a matricellular protein associated with increased fibrosis and glaucoma pathogenesis [55]. Studies have shown that SPARC-null mice have reduced IOP [55] and overexpression of SPARC in the TM of perfused cadaveric human anterior segments increases IOP [56], [57]. To determine whether laser photocoagulation induces inflammatory response and fibrotic changes in TM tissue resulting in IOP elevation, the expression of inflammatory cell marker CD45 and fibrotic marker SPARC in laser-treated and control eyes was examined using cryosections and wholemount tissues. CD45 inflammatory cells increased dramatically in the TM region 1 week after laser (Fig 7). The expression of SPARC increased in the TM region beginning 1 week after laser treatment (Fig 6). This finding is consistent with previous discoveries that SPARC has a regulatory role in IOP [56]. We speculate that inflammatory response after laser plays a role in promoting the fibrotic changes of the ECM and the combined changes of TM cellularity and ECM after laser photocoagulation are the main cause for IOP elevation.

The functional morphology of the mouse outflow pathway is similar to that of humans. The mouse eye resembles the human eye not only in the presence of a continuous Schlemm's canal and comparable TM, but also in the three dimensional elastic fiber network connected to the inner wall, to ciliary muscle and to choroidal vessels. All these are very important to control aqueous outflow (Lutjen-Drecoll E, et al. *IOVS* 2013; 54: ARVO E-Abstract 3544). Our mouse model produces the pathological changes which block the outflow pathway in a similar way to that found in glaucomatous humans. It induces long-lasting IOP elevation, outflow structural changes including disorganized ECM (Fig 8), open anterior chamber angle, loss of RGC axons and decreased PhNR, mirroring all the characteristics of human POAG. Using stem cells to reconstruct the TM in glaucomatous eyes is one of the potential therapy strategies for POAG. We previously demonstrated that adult stem cells have an ability to remodel tissue matrix [39]. We also reported that stem cells from human TM can home to the TM region after being transplanted into normal mouse anterior chamber [38]. We hypothesize that once the decreased TM cellularity in glaucomatous eye is repopulated and the ECM is remodeled after stem cell transplantation, the outflow facility will be restored which would reduce the increased IOP back to normal. A suitable animal model for cell-based therapies requires TM cellular damage, outflow pathway ECM changes with open anterior chamber angle from which exogenous stem cells can access and target to the TM. We therefore speculate that this mouse glaucoma model could be valuable for studies of TM stem cell-based therapies for glaucoma on reconstructing the outflow pathway both morphologically and functionally. Work is ongoing to determine the ability of adult stem cells to home to damaged TM tissue and remodel the tissue to regulate IOP.
